# Higher serum free thyroxine levels are associated with increased risk of hip fractures in older men

**DOI:** 10.1093/jbmr/zjad005

**Published:** 2024-01-04

**Authors:** Johan Svensson, Claes Ohlsson, Magnus K Karlsson, Hans Herlitz, Mattias Lorentzon, Catharina Lewerin, Dan Mellström

**Affiliations:** Department of Internal Medicine and Clinical Nutrition, Sahlgrenska Osteoporosis Center, Center for Bone and Arthritis Research, Institute of Medicine, Sahlgrenska Academy, University of Gothenburg, Gothenburg SE-413 45, Sweden; Department of Internal Medicine, Region Västra Götaland, Skaraborg Central Hospital, Skövde SE-541 85, Sweden; Department of Internal Medicine and Clinical Nutrition, Sahlgrenska Osteoporosis Center, Center for Bone and Arthritis Research, Institute of Medicine, Sahlgrenska Academy, University of Gothenburg, Gothenburg SE-413 45, Sweden; Department of Drug Treatment, Region Västra Götaland, Sahlgrenska University Hospital, Gothenburg SE-413 45, Sweden; Clinical and Molecular Osteoporosis Research Unit, Department of Clinical Sciences and Orthopedics, Skane University Hospital (SUS), Lund University, Malmö SE-205 02, Sweden; Department of Molecular and Clinical Medicine/Nephrology, Institute of Medicine, Sahlgrenska Academy, University of Gothenburg, Gothenburg SE-413 45, Sweden; Department of Internal Medicine and Clinical Nutrition, Sahlgrenska Osteoporosis Center, Center for Bone and Arthritis Research, Institute of Medicine, Sahlgrenska Academy, University of Gothenburg, Gothenburg SE-413 45, Sweden; Geriatric Medicine, Department of Internal Medicine and Clinical Nutrition, Institute of Medicine, Sahlgrenska Academy, University of Gothenburg, Gothenburg SE-413 45, Sweden; Mary MacKillop Institute for Health Research, Australian Catholic University, Melbourne, VIC, 3000, Australia; Department of Hematology and Coagulation, Region Västra Götaland, Sahlgrenska University Hospital, Gothenburg SE-413 45, Sweden; Department of Internal Medicine and Clinical Nutrition, Institute of Medicine, Sahlgrenska Academy, University of Gothenburg, Gothenburg SE-413 45, Sweden; Department of Internal Medicine and Clinical Nutrition, Sahlgrenska Osteoporosis Center, Center for Bone and Arthritis Research, Institute of Medicine, Sahlgrenska Academy, University of Gothenburg, Gothenburg SE-413 45, Sweden; Geriatric Medicine, Department of Internal Medicine and Clinical Nutrition, Institute of Medicine, Sahlgrenska Academy, University of Gothenburg, Gothenburg SE-413 45, Sweden

**Keywords:** serum free thyroxine, serum thyroid-stimulating hormone, fracture risk, older men, hip fracture

## Abstract

Overt and subclinical hyperthyroidism are associated with an increased fracture risk, but whether thyroid hormones are associated with fracture risk in individuals with normal thyroid-stimulating hormone (TSH) has mostly been investigated in women. Therefore, we investigated if serum levels of free thyroxine (FT4) or TSH are associated with fracture risk in Swedish men. We followed (median 12.2 yr) elderly men (*n* = 1825; mean age 75, range 69–81 yr) participating in the Gothenburg and Malmö subcohorts of the prospective, population-based MrOS-Sweden study. The statistical analyses included Cox proportional hazards regression. Men receiving levothyroxine treatment were excluded. In our total cohort, serum FT4 (per SD increase) was associated with increased risk of major osteoporotic fractures (MOFs; *n* = 479; fully adjusted hazard ratio [HR] 1.14, 95% CI, 1.05–1.24) and hip fractures (*n* = 207; HR 1.18, 95% CI, 1.04–1.33). Also, in men with normal TSH (*n* = 1658), FT4 (per SD increase) was significantly associated with increased risk of MOF and hip fractures. Furthermore, men in the highest FT4 quartile had a 1.5-fold increase in hip fracture risk compared with men in the three lower FT4 quartiles, both in the total population and in men with normal TSH (fully adjusted: HR 1.45, 95% CI, 1.04–2.02 and HR 1.51, 95% CI, 1.07–2.12, respectively). In contrast, the risk of MOF was not statistically different in the highest FT4 quartile compared with the three lower FT4 quartiles. Finally, serum TSH was not associated with fracture risk after full adjustment for covariates. In conclusion, serum FT4, but not serum TSH, is a predictor of hip fracture risk in elderly Swedish men. Additionally, there was an association between FT4 (per SD increase) and the risk of MOF.

## Teaser

Thyroid hormone excess (hyperthyroidism) is associated with increased fracture risk. However, whether thyroid hormones are associated with fracture risk in individuals with normal thyroid-stimulating hormone (TSH), which regulates free thyroxine (FT4), has mostly been investigated in women. Therefore, we investigated if FT4 or TSH levels are associated with fracture risk in Swedish men. We followed (median 12.2 yr) 1825 elderly men (mean age 75 yr) in the population-based MrOS-Sweden study. During the follow-up, 479 men suffered from a major osteoporotic fracture (MOF) and 207 men sustained a hip fracture. In our total cohort as well as in men with normal TSH, serum FT4 (per SD increase) was associated with increased risk of MOF and hip fractures. Furthermore, men in the highest FT4 quartile had a 1.5-fold increase in hip fracture risk compared with men in the three lower FT4 quartiles, both in the total population and in men with normal TSH. In contrast, the risk of MOF was similar in the highest FT4 quartile compared with the three lower quartiles. Finally, serum TSH was not associated with fracture risk. In conclusion, serum FT4, but not serum TSH, is a predictor of hip fracture risk in elderly Swedish men.

## Introduction

In adult life, untreated overt hyperthyroidism results in secondary osteoporosis and increased fracture risk.^1^ Furthermore, overtreatment with levothyroxine results in reduced BMD and increased risk of fractures.^1–4^ There is also some evidence that suppressed thyroid-stimulating hormone (TSH) with free thyroxine (FT4) and free triiodothyronine levels within the normal range, denominated subclinical hyperthyroidism (SHyper), is associated with impaired bone health.^5^^,^^6^ Although the results of individual studies are varying,^7–11^ a recent analysis of the Atherosclerosis Risk in Communities Study (*n* = 10 946, 54.3% women) showed a moderately increased fracture risk in SHyper classified as TSH < 0.56 mIU/L.^12^ Furthermore, a meta-analysis demonstrated that SHyper, defined as TSH < 0.45 mIU/L, was associated with increased risk of any fracture and hip fracture, whereas there were only tendencies to increased risks of spine and non-spine fractures.^5^ In another meta-analysis, in which the cut-off TSH value for SHyper varied between 0.30 and 0.55 mIU/L, there were moderate but significant associations between SHyper and increased risk of any, hip, spine, and non-spine fractures.^6^ Finally, SHyper was moderately associated with increased fracture risk in a systematic review and meta-analysis.^13^

Studies in postmenopausal women and in populations comprising both men and women have shown inconsistent results whether there is an association between TSH or FT4 levels and fracture risk in individuals with normal TSH. In postmenopausal women (*n* = 2374), serum FT4 was positively and serum TSH was negatively associated with the risk of nonvertebral fractures,^14^ whereas another study (*n* = 533) demonstrated lower TSH values at a 5-yr follow-up in women with incident major osteoporotic fractures (MOFs).^15^ In the Hunt cohort (16 610 women and 8595 men), there was no association between baseline serum TSH and the risk of hip or forearm fractures.^16^ In a one-sample Mendelian randomization study (*n* = 5599), genetically raised serum TSH was causally associated with decreased risk of an osteoporotic fracture in men but not in women.^17^ In subjects with normal TSH in a Danish register-based study, the risks of hip fractures and MOF were increased with each SD unit of TSH decrease.^18^ In a pooled analysis of 13 prospective cohort studies, in adults with baseline TSH of 0.45–4.49 mIU/L, lower TSH levels as well as higher FT4 levels were associated with increased risk of hip fractures.^19^

Previously, three prospective studies have been performed only in men. In a study by Siru et al. (*n* = 3338), neither TSH nor FT4 levels were predictive of hip fracture risk.^20^ However, in the MrOS-US study, reduced serum TSH (per SD decrease) was borderline associated with increased risk of hip fractures in men with normal TSH (0.55–4.78 mIU/L).^21^ In an earlier study based on MrOS-Sweden (*n* = 1856) with shorter median follow-up (9.8 vs 12.2 yr in the current study) and fewer incident hip fractures (*n* = 127 vs *n* = 207 in the present study), TSH and FT4 levels were not associated with the risk of hip or vertebral fractures in the entire study population, whereas the risk of vertebral fractures was increased in men with TSH < 0.45 mIU/L.^22^

In summary, overt hyperthyroidism and SHyper have been associated with increased fracture risk.^5^^,^^6^ However, individual studies have produced varying results, and there is no consensus whether there is an independent association between thyroid hormones and facture risk in subjects with normal TSH or whether the risk of fractures is different in men and women. In the present study, we evaluated whether serum FT4 and TSH levels in elderly men are associated with fracture risk in two well-controlled subcohorts of the prospective, population-based MrOS-Sweden study with access to validated fracture data based on computerized X-ray archives. We hypothesized that lower TSH and higher FT4 levels would be associated with increased fracture risk in the total study population as well as in men with normal serum TSH.

## Materials and methods

### Participants and ethical considerations

The Osteoporotic Fractures in Men Study (MrOS) is an international, multicenter study of older men. The present observational study involved the Malmö (*n* = 1005) and Gothenburg (*n* = 1010) subcohorts of MrOS-Sweden. In MrOS-Sweden, men aged 69–81 yr were contacted and asked to participate after having been randomly identified using national population registers.^23^ Inclusion criteria were ability to walk without assistance, provide informed consent, and to be able to supply self-reported information. There were no other inclusion/exclusion criteria. Of the men contacted, 45% were included in the study. At baseline (2001–2004), the men were physically examined and answered a questionnaire. In addition, at baseline, blood samples were obtained and BMD measurements were performed (see below). Then, the men were followed until August 2018, and all new fractures that occurred during the follow-up were recorded.

Serum TSH was measured in 1856 of the elderly men (92% of the total cohort). However, we excluded one man with previous history of thyroid cancer and 31 additional men receiving levothyroxine treatment at baseline, which resulted in a study population of 1825 men. Serum FT4 had been measured in 1746 of these men. In all men (*n* = 1825 with serum TSH) as well as in the subpopulation of men with normal serum TSH (*n* = 1658), we investigated whether serum concentrations of TSH or FT4 were associated with the risk of fractures. We defined a normal TSH as serum TSH between 0.45 mIU/L and < 4.5 mIU/L^5^^,^^19^^,^^24^ and SHyper as serum TSH < 0.45 mIU/L combined with serum FT4 ≤ 22 pmol/L (the upper normal limit of the reference range). Subclinical hypothyroidism (SHypo) was defined as serum TSH ≥ 4.5 mIU/L^5^^,^^19^^,^^24^ combined with serum FT4 in the normal range (12–22 pmol/L).

Approval of the study had been received from the ethics committees at Lund University (LU 693/00) and Gothenburg University (Gbg M 014–1). The study was performed in accordance with the 1964 Helsinki declaration and its later amendments.

### Determination of BMD

DXA was used to assess areal BMD (g/cm^2^; later referred to as BMD). In Malmö, the Lunar Prodigy DXA (GE Lunar Corp., Madison, WI) was employed and in Gothenburg, the Hologic QDR 4500/A-Delphi (Hologic, Waltham, MA) was applied. Because DXA was performed differently in the two centers, standardized BMD (sBMD) values for total hip and lumbar spine were calculated as described earlier.^23^ The BMD assessments had coefficients of variation (CVs) between 0.5% and 3%.

### Covariate assessment

Standardized measurements of body height and weight were performed. BMI was determined by dividing weight (in kilograms) with height (in meters) squared. Appendicular lean mass was assessed using the Lunar Prodigy DXA (GE Lunar Corp.) in Malmö and using the Hologic QDR 4500/A-Delphi (Hologic, Inc.) in Gothenburg. Hand grip strength was determined using a Jamar® hand dynamometer (Jackson, MI)^25^; we used the maximum value (kilograms) from two trials of both hands. A standardized questionnaire was employed to obtain information of current smoking (yes / no). A cystatin C-based formula was applied to estimate glomerular filtration rate (GFR).^26^

### Biochemical methods

All blood samples were collected at baseline (from 2001 to 2004), and the obtained serum was stored at −80 ^°^C pending analysis. In the Gothenburg cohort, serum was drawn in the fasted state at 8:00 AM. The serum samples in the Malmö cohort were collected before 10:00 AM (40% of the Malmö cohort) or in the non-fasted state around noon (between 10:00 AM and 3:00 PM; 60% of the Malmö cohort). Cystatin C in serum (used to calculate estimated GFR as stated above) was determined by the Hitachi Modular P analyzer with reagents and calibrators from Dako A/S (Copenhagen, Denmark).^26^ FT4 and TSH in serum were analyzed in 2008 on the Roche Modular system (Roche Diagnostics Scandinavia AB, Solna, Sweden).^27^ The total CV was below 10% for both the FT4 assay and the TSH assay. The detection limits were 1.5 pmol/L for the FT4 assay and 0.005 mIU/L for the TSH assay.

### Fracture assessment

The average follow-up time was 10.9 (SD 4.4) yr (median 12.2 yr, 25th–75th percentiles: 7.5–14.8 yr). We defined the follow-up time as the time between the date of the baseline visit and the occurrence of fracture, death, or study end (August 31, 2018). In the present study, the follow-up time was ended due to death in 789 of the participants. In comparison, in the earlier study of the association between thyroid hormones and fracture risk based on MrOS-Sweden,^22^ the reason for discontinuation of follow-up was death in 530 men. All fractures were recorded during the follow-up, and when subjects suffered from a first fracture at all sites, the follow-up time for respective fracture was analyzed. Data in terms of deaths were received from the National Cause of Death Register in which all deaths in Sweden are recorded.

The time and site of fracture were received from computerized X-ray archives by using the unique personal registration number that all Swedish citizens have. A vertebral fracture was defined as the participant-reported clinical symptoms after the baseline visit and confirmation of a vertebral fracture by physician review of the radiology report. Thus, all the included fractures (vertebral and others) were verified by physician review of the radiology reports. Participant-reported fractures that could not be confirmed by radiology were excluded. In the present study, we analyzed all the validated fractures, hip fractures, vertebral fractures, and MOFs. MOF was defined as the fracture of the hip, pelvis, proximal humerus, forearm, and vertebral fractures.

### Statistical analyses

We used SPSS for Windows (version 28.0, IBM Corp., Armonk, NY). Data are presented as the mean and SD if not otherwise stated. Differences between groups were assessed using one-way ANOVA for continuous variables and using chi-square tests for categorical variables.

Cox proportional hazards regression analyses were used to calculate hazard ratios (HRs) and 95% CIs for the associations between serum concentrations of TSH and FT4 and the risk of fractures. In the entire study population (*n* = 1825 with serum TSH) as well as in the subpopulation of men having normal serum TSH (*n* = 1658), we determined whether FT4 and TSH as standardized continuous variables (per SD increase in serum FT4 and per SD decrease in serum TSH) were associated with the risk of all fractures, MOF, hip fractures, or vertebral fractures. In all analyses, we adjusted for age and MrOS site. Furthermore, to evaluate the independent effect of FT4 or TSH on fracture risk, additional adjustments were made for BMI, appendicular lean mass, hand grip strength, estimated GFR, and current smoking (model A). In model B, total hip sBMD was also included as a covariate. In addition, we analyzed the risk of fractures in the highest FT4 quartile (quartile IV) compared with that in the three lower quartiles (quartiles I–III) using Cox proportional hazards regression. Finally, we performed a subanalysis in which we added time of serum sampling (before 10:00 AM; yes/no) as a covariate in the previously performed Cox proportional hazards regression analyses. A two-sided *P* < .05 was considered statistically significant.

## Results

### Baseline characteristics

The baseline characteristics in the total cohort and by incident hip or vertebral fracture status are given in Table 1. Men who sustained a hip fracture were older and had lower grip strength than men not suffering from an incident hip fracture, whereas men who had an incident vertebral fracture had lower appendicular lean mass than men free of vertebral fractures. In men with a hip fracture as well as in men with a vertebral fracture, lumbar spine L1–L4 sBMD and total hip sBMD were lower than in the groups not having these fractures. Serum TSH and FT4 were statistically similar in the fracture groups compared with the fracture-free groups.

**Table 1:** Baseline characteristics in the total study population and by incident hip or vertebral fracture status.

Variable	All men	Incident hip fracture		*P* ^a^	Incident vertebral fracture		*P* ^a^
	*n* = 1825	Yes, *n* = 207	No, *n* = 1618		Yes, *n* = 249	No, *n* = 1576	
Age (yr)	75.4 (3.2)	75.8 (3.1)	75.3 (3.2)	<.05	75.4 (3.3)	75.4 (3.2)	.97
BMI (kg/m^2^)	26.4 (3.6)	26.2 (3.6)	26.4 (3.6)	.41	26.1 (3.7)	26.4 (3.6)	.22
Appendicular lean mass (kg)	24.6 (3.6)	24.3 (3.2)	24.6 (3.3)	.18	24.1 (3.3)	24.6 (3.3)	<.05
Grip strength (kg)	43.2 (8.0)	42.0 (7.7)	43.4 (8.0)	<.05	42.4 (7.6)	43.4 (8.0)	.07
Estimated GFR (mL/min/ 1.73 m^2^)	70.4 (19.4)	69.9 (18.4)	70.4 (19.4)	.66	70.4 (23.4)	70.4 (18.7)	.99
Current smoking (yes / no), % (*n*)	9/91 (165/1660)	10/90 (20/187)	9/91 (145/1473)	.74	12/88 (29/220)	9/91 (136/1440)	.27
Lumbar spine L1 to L4 sBMD (g/cm^2^)	1.13 (0.19)	1.08 (0.17)	1.13 (0.20)	<.001	1.04 (0.17)	1.14 (0.19)	<.001
Total hip sBMD (g/cm^2^)	0.94 (0.14)	0.86 (0.12)	0.95 (0.14)	<.001	0.89 (0.13)	0.95 (0.14)	<.001
Serum TSH (mIU/L)	2.60 (3.61)	2.48 (3.44)	2.61 (3.64)	.60	2.31 (1.20)	2.64 (3.86)	.18
Serum FT4 (pmol/L)	17.1 (3.5)	17.4 (2.8)	17.0 (3.5)	.21	17.2 (2.9)	17.0 (3.6)	.61

^a^*P*-values between men having or not having an incident fracture of the given type

If not otherwise stated, values are given as means (SD). Between-group differences were examined using one-way analysis of variance (ANOVA) for continuous variables and using chi-square tests for categorical variables.

BMI = Body mass index; FT4 = free thyroxine; GFR = glomerular filtration rate; sBMD = standardized bone mineral density; TSH = thyroid-stimulating hormone.

### SHyper and SHypo

We only found 22 men who suffered from SHyper (serum TSH < 0.45 mIU/L and serum FT4 ≤ 22 pmol/L), and no more than three of these men sustained an incident hip fracture. Therefore, we did not perform further analyses in men with SHyper. Furthermore, we also did not perform additional analyses in the 125 men with SHypo (serum TSH ≥ 4.5 mIU/L and serum FT4 = 12–22 pmol/L; 11 incident hip fractures).

### Serum FT4 and fracture risk

During follow-up (mean 10.9 [SD 4.4] yr, median 12.2 [25th–75th percentiles: 7.5–14.8] yr), 622 (34%) of the men suffered from an incident fracture. In all men having measurement of serum FT4 (*n* = 1746), Cox proportional hazards regression was used to analyze whether serum FT4 as a standardized continuous variable was associated with fracture risk. Serum FT4 (per SD increase) was associated with the risk of all fractures in model A, but not in the base model or model B (Table 2). In contrast, higher serum FT4 was associated with increased risk of MOF and hip fractures both in the base model and the adjusted models (model B: HR 1.14, 95% CI, 1.05–1.24 and HR 1.18, 95% CI, 1.04–1.33, respectively) (Table 2). Serum FT4 was not associated with the risk of vertebral fractures in the base model, but in the adjusted models, higher serum FT4 was significantly associated with increased vertebral fracture risk (model B: HR 1.14, 95% CI, 1.02–1.28; Table 2).

**Table 2:** Risk (hazard ratios and 95% CIs) of first fracture per SD increase in serum FT4 levels.

	Total study population (*n* = 1746)^a^	Men with normal serum TSH (*n* = 1585)^b^
*All fractures*		
Fractures, *n* (%)	595 (34%)	543 (34%)
Base model	1.07 (0.98–1.17)	1.10 (1.01–1.19)
Multivariate model A	1.10 (1.01–1.19)	1.09 (0.99–1.19)
Multivariate model B	1.09 (0.99–1.20)	1.08 (0.97–1.19)
*Major osteoporotic fractures*		
Fractures, *n* (%)	457 (26%)	415 (26%)
Base model	1.12 (1.03–1.21)	1.13 (1.05–1.22)
Multivariate model A	1.14 (1.05–1.23)	1.13 (1.04–1.22)
Multivariate model B	1.14 (1.05–1.24)	1.13 (1.03–1.23)
*Hip fractures*		
Fractures, *n* (%)	194 (11%)	179 (11%)
Base model	1.17 (1.07–1.29)	1.17 (1.06–1.29)
Multivariate model A	1.17 (1.06–1.30)	1.17 (1.05–1.30)
Multivariate model B	1.18 (1.04–1.33)	1.17 (1.04–1.32)
*Vertebral fractures*		
Fractures, *n* (%)	241 (14%)	218 (14%)
Base model	1.11 (0.99–1.25)	1.13 (1.02–1.25)
Multivariate model A	1.15 (1.03–1.27)	1.12 (0.99–1.27)
Multivariate model B	1.14 (1.02–1.28)	1.11 (0.97–1.28)

Hazard ratios were calculated using Cox proportional hazards regression, in which serum FT4 was included as a standardized continuous variable (per SD increase).

^a^Men with measurement of serum FT4

^b^Men with measurement of FT4 and serum TSH between 0.45 and < 4.5 mIU/L

Base model: adjustment for age and MrOS site

Multivariate model A: age, MrOS site, BMI, appendicular lean mass, grip strength, estimated GFR, and current smoking (yes / no)

Multivariate model B: age, MrOS site, BMI, appendicular lean mass, grip strength, estimated GFR, current smoking, and total hip sBMD.

In men with normal serum TSH (TSH between 0.45 and < 4.5 mIU/L; *n* = 1585 with serum FT4 measurement), there was a significant association between serum FT4 (per SD increase) and the risk of all fractures in the base model (HR 1.10, 95% CI, 1.01–1.19), but not in the adjusted models (Table 2). Furthermore, higher FT4 was associated with increased risk of MOF and hip fractures, and these associations were retained in all the adjusted models (model B: HR 1.13, 95% CI, 1.03–1.23 and HR 1.17, 95% CI, 1.04–1.32, respectively) (Table 2). Finally, FT4 was associated with the risk of vertebral fractures in the base model (HR 1.13, 95% CI, 1.02–1.25), but not in the adjusted models (Table 2).

### Analyses of men in the highest serum FT4 quartile

Next, we compared the highest FT4 quartile (quartile IV) with the three lower FT4 quartiles (quartiles I–III). At baseline (*n* = 1746), the clinical characteristics were similar in quartile IV and quartiles I–III except for current smoking, which was more common in the highest FT4 quartile (*n* = 47 [12%]) compared with the lower three quartiles (*n* = 110 [8%]) (*P* < .05, data not shown). There were no differences in lumbar spine L1–L4 sBMD (quartile IV: mean 1.12 [SD 0.20] g/cm^2^ vs quartiles I–III: 1.13 [0.19] g/cm^2^; *P* = .52) or total hip sBMD (both groups: mean 0.94 g/cm^2^; *P* = .59).

**Figure 1:**
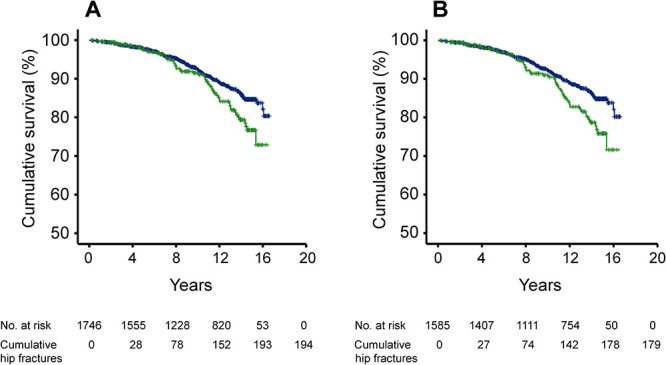
Higher serum FT4 is associated with increased risk of incident hip fractures. Kaplan–Meier survival curves for the risk of hip fractures in (A) all men with measurement of serum FT4 (*n* = 1746; log-rank test: *P* = .02), and (B) men with normal serum TSH (between 0.45 and < 4.5 mIU/L) and measurement of serum FT4 (*n* = 1585; log-rank test: *P* < .01). *Green*, men in the highest FT4 quartile (quartile IV), *blue*, men in the three lower FT4 quartiles (quartiles I–III).

Cox proportional hazards regression analyses showed that in all men with serum FT4 (*n* = 1746), the risk of all fractures and vertebral fractures was similar in the highest FT4 quartile (quartile IV) as that in in the lower quartiles (quartiles I–III) (Table 3). The risk of MOF was increased in the highest FT4 quartile in the base model (vs quartiles I-III: HR 1.26, 95% CI, 1.02–1.55), but not in the adjusted models. In contrast, there was a marked increase in the risk of hip fractures in the highest FT4 quartile compared with the lower three quartiles (base model: HR 1.46, 95% CI, 1.07–2.00; model B: HR 1.45, 95% CI, 1.04–2.02) (Table 3). Kaplan–Meier survival curves further illustrated that the highest FT4 quartile was associated with increased hip fracture risk (log-rank test, *P* = .02; Figure 1A).

**Table 3:** Risk (hazard ratios and 95% CIs) of first fracture in the highest serum FT4 quartile compared with the three lower FT4 quartiles.

	Total study population (*n* = 1746)^a^		Men with normal serum TSH (*n* = 1585)^b^	
	Quartile IV (*n* = 402) (>75th percentile)	Quartiles I–III (*n* = 1344) (≤75th percentile)	Quartile IV (*n* = 368) (>75th percentile)	Quartiles I–III (*n* = 1217) (≤75th percentile)
*All fractures*				
Fractures, *n* (%)	146 (36%)	449 (33%)	135 (37%)	408 (34%)
Base model	1.18 (0.97–1.42)	1.0 referent	1.20 (0.99–1.46)	1.0 referent
Multivariate model A	1.14 (0.93–1.39)	1.0 referent	1.12 (0.91–1.37)	1.0 referent
Multivariate model B	1.13 (0.92–1.38)	1.0 referent	1.11 (0.90–1.36)	1.0 referent
*Major osteoporotic fractures*				
Fractures, *n* (%)	117 (29%)	340 (25%)	109 (30%)	306 (25%)
Base model	1.26 (1.02–1.55)	1.0 referent	1.31 (1.05–1.64)	1.0 referent
Multivariate model A	1.23 (0.99–1.54)	1.0 referent	1.24 (0.98–1.56)	1.0 referent
Multivariate model B	1.22 (0.98–1.53)	1.0 referent	1.22 (0.97–1.55)	1.0 referent
*Hip fractures*				
Fractures, *n* (%)	55 (14%)	139 (10%)	52 (14%)	127 (10%)
Base model	1.46 (1.07–2.00)	1.0 referent	1.54 (1.11–2.13)	1.0 referent
Multivariate model A	1.46 (1.05–2.02)	1.0 referent	1.50 (1.07–2.10)	1.0 referent
Multivariate model B	1.45 (1.04–2.02)	1.0 referent	1.51 (1.07–2.12)	1.0 referent
*Vertebral fractures*				
Fractures, *n* (%)	59 (15%)	182 (14%)	53 (14%)	165 (14%)
Base model	1.17 (0.87–1.57)	1.0 referent	1.16 (0.85–1.59)	1.0 referent
Multivariate model A	1.10 (0.81–1.51)	1.0 referent	1.05 (0.75–1.45)	1.0 referent
Multivariate model B	1.09 (0.80–1.49)	1.0 referent	1.03 (0.74–1.44)	1.0 referent

Hazard ratios were calculated using Cox proportional hazards regression.

^a^Men with measurement of serum FT4

^b^Men with measurement of FT4 and serum TSH between 0.45 and < 4.5 mIU/L

Base model: adjustment for age and MrOS site

Multivariate model A: age, MrOS site, BMI, appendicular lean mass, grip strength, estimated GFR, and current smoking (yes / no)

Multivariate model B: age, MrOS site, BMI, appendicular lean mass, grip strength, estimated GFR, current smoking, and total hip sBMD.

In men with normal TSH (*n* = 1585 with serum FT4), there was also a prominent increase in the risk of hip fractures in the highest FT4 quartile compared with the lower three quartiles (base model: HR 1.54, 95% CI, 1.11–2.13; model A: HR 1.50, 95% CI, 1.07–2.10; model B: HR 1.51, 95% CI, 1.07–2.12) (Table 3). In addition, the risk of MOF was increased in the base model (quartile IV vs quartile I–III: HR 1.31, 95% CI, 1.05–1.64). Otherwise, there were no significant differences between the highest FT4 quartile and the three lower quartiles in the risk of all fractures, MOF, or vertebral fractures (Table 3). Finally, Kaplan–Meier survival curves confirmed that the highest FT4 quartile was associated with increased hip fracture risk in men with normal TSH (log-rank test, *P* < .01; Figure 1B).

### Serum TSH and fracture risk

In all men (*n* = 1825), Cox proportional hazards regression showed that serum TSH as a standardized continuous variable (per SD decrease) was not associated with the risk of all fractures, MOF, hip fractures, or vertebral fractures (Table 4). Furthermore, in men having normal TSH (*n* = 1658), there was no significant association between serum TSH (per SD decrease) and the risk of hip fractures in the base model (Table 4). However, serum TSH (per SD decrease) was significantly associated with increased hip fracture risk in model A (HR 1.17, 95% CI, 1.002–1.36), which did not remain significant after additional adjustment for total hip sBMD in model B (HR 1.11, 95% CI, 0.96–1.30) (Table 4).

**Table 4:** Risk (hazard ratios and 95% CIs) of first fracture per SD decrease in serum TSH levels.

	Total study population (*n* = 1825)	Men with normal serum TSH (*n* = 1658)^a^
*All fractures*		
Fractures, *n* (%)	622 (34%)	567 (34%)
Base model	1.10 (0.96–1.27)	1.07 (0.99–1.17)
Multivariate model A	1.13 (0.96–1.32)	1.08 (0.99–1.18)
Multivariate model B	1.08 (0.94–1.24)	1.04 (0.96–1.14)
*Major osteoporotic fractures*		
Fractures, *n* (%)	479 (26%)	434 (26%)
Base model	1.14 (0.95–1.36)	1.04 (0.95–1.15)
Multivariate model A	1.12 (0.94–1.34)	1.05 (0.95–1.16)
Multivariate model B	1.07 (0.92–1.25)	1.01 (0.91–1.11)
*Hip fractures*		
Fractures, *n* (%)	207 (11%)	192 (12%)
Base model	1.07 (0.86–1.32)	1.15 (0.996–1.34)
Multivariate model A	1.07 (0.87–1.32)	1.17 (1.002–1.36)
Multivariate model B	1.01 (0.85–1.20)	1.11 (0.96–1.30)
*Vertebral fractures*		
Fractures, *n* (%)	249 (14%)	224 (14%)
Base model	1.33 (0.96–1.85)	0.95 (0.83–1.09)
Multivariate model A	1.31 (0.93–1.83)	0.97 (0.84–1.11)
Multivariate model B	1.23 (0.89–1.69)	0.93 (0.82–1.07)

Hazard ratios were calculated using Cox proportional hazards regression, in which serum TSH was included as a standardized continuous variable (per SD decrease).

^a^Men with serum TSH between 0.45 and < 4.5 mIU/L

Base model: adjustment for age and MrOS site

Multivariate model A: age, MrOS site, BMI, appendicular lean mass, grip strength, estimated GFR, and current smoking (yes / no)

Multivariate model B: age, MrOS site, BMI, appendicular lean mass, grip strength, estimated GFR, current smoking, and total hip sBMD.

### Subanalyses

At baseline, 31 men received treatment with either oral glucocorticoids (*n* = 23), bisphosphonates (*n* = 7), or both oral glucocorticoids and bisphosphonates (*n* = 1). Additional analyses in the total study population (*n* = 1746 with measurement of FT4) showed that after exclusion of the 31 men treated with oral glucocorticoids and/or bisphosphonates, FT4 (per SD increase) was still associated with the risk of MOF (model B: HR 1.14, 95% CI, 1.04–1.24), hip fractures (model B: HR 1.17, 95% CI, 1.03–1.33), and vertebral fractures (model B: HR 1.14, 95% CI, 1.02–1.28) after full adjustment for covariates. Furthermore, in men with normal TSH (*n* = 1585 with serum FT4), FT4 (per SD increase) was still associated with the risk of MOF (model B: HR 1.13, 95% CI, 1.03–1.24) and hip fractures (model B: HR 1.17, 95% CI, 1.03–1.32) after excluding those treated with oral glucocorticoids and/or bisphosphonates. Finally, men in the highest FT4 quartile (quartile IV) had increased risk of hip fractures compared with those in the three lower quartiles (quartile I–III) both in the total study population (model B: HR 1.43, 95% CI, 1.03–2.00) and in men with normal TSH (model B: HR 1.49, 95% CI, 1.05–2.10) even after exclusion of the men treated with glucocorticoids and/or bisphosphonates.

Finally, as there could be diurnal variations in FT4 levels, we performed subanalyses in which we additionally adjusted for time of serum sampling (before 10:00 AM; yes/no). For the associations between serum FT4 and the risks of MOF and hip fractures, all the observed associations remained significant even when the time of serum sampling was added as an additional covariate (data not shown). However, the association between FT4 (per SD increase) and the risk of vertebral fractures in the total study population marginally lost statistical significance (model B and additional adjustment for time of serum sampling: HR 1.12, 95% CI, 0.99–1.28).

## Discussion

Overt hyperthyroidism and SHyper have been associated with an increased fracture risk, but there is no consensus on whether there is an independent association between thyroid hormones and facture risk in elderly men with normal TSH. In the present study, we evaluated the association between serum thyroid hormone levels and fracture risk in two of the well-controlled MrOS-Sweden subcohorts with reliable fracture data based on computerized X-ray archives. We excluded men receiving levothyroxine treatment and one man with previous history of thyroid cancer, and we performed analyses in the total cohort as well in men with normal serum TSH. We found that higher serum FT4, as a standardized continuous variable, was significantly associated with increased risk of hip fractures. In addition, men in the highest serum FT4 quartile had a 1.5-fold increase in the risk of hip fractures compared with men in the three lower FT4 quartiles. Moreover, serum FT4 levels (per SD increase) were associated with the risk of MOF, but there was only a tendency to an increased risk of MOF in the highest serum FT4 quartile compared with the three lower quartiles. Finally, serum TSH levels were not associated with the risk of fractures after full adjustment for covariates.

A major finding of the present study is the observed association between serum FT4 levels (per SD increase) and increased hip fracture risk both in our total cohort as well as in men with normal serum TSH (TSH between 0.45 mIU/L and < 4.5 mIU/L). Additionally, men in the highest serum FT4 quartile had a marked (1.5-fold) increase in the risk of hip fractures compared with men in the three lower quartiles. In terms of the clinical characteristics at baseline, patients suffering from an incident hip fracture had higher age, lower grip strength, and lower BMD compared with other men. However, we adjusted for these and multiple other covariates in the statistical analyses, and all associations between FT4 and hip fracture risk remained significant in the fully adjusted analyses. Therefore, the mechanisms underlying the association between higher serum FT4 levels and increased hip fracture risk are not fully clear, but we cannot exclude the possibility that higher FT4 levels induced changes in bone turnover or musculoskeletal function that were not detected by the tests used in our study.

In the present study, the Kaplan–Meier survival curves illustrated that the cumulative risk of hip fractures in the highest FT4 quartile gradually deviated from that in three lower FT4 quartiles. However, the difference between the curves was relatively small during the first 6–8 yr of follow-up, which could be due to the relatively low number of incident hip fractures during the first years of our study. On the other hand, it is possible that a relatively long duration of highnormal FT4 is required before the risk of hip fracture starts to increase. Anyhow, our results show that serum FT4 is a strong predictor of the long-term risk of hip fractures. Therefore, preventive actions to counteract the consequences of high serum FT4 can possibly be initiated when the risk of hip fractures is still moderate. One example could be low-risk differentiated thyroid cancer, a condition in which suppressive doses of levothyroxine increased the risk of postoperative osteoporosis without any effect on tumor recurrence.^28^ Therefore, the avoidance of therapeutic harm should be a major aim of levothyroxine therapy in low-risk thyroid cancer patients.^1^^,^^28^

The present study (*n* = 1825, mean age 75 yr) had a longer duration of follow-up (median 12.2 yr) and more incident hip fractures (*n* = 207) compared with the three previous studies that have explored the association between thyroid hormones and fracture risk in study populations consisting only of older men. Siru et al. did not find any association between FT4 or TSH levels and the risk of incident hip fractures (*n* = 3338, mean age 77 yr, 161 hip fractures, median follow-up not given but the maximum follow-up was from year 2001 to the end of year 2012).^20^ In the MrOS-US cohort (*n* = 1817, mean age 74 yr, mean follow-up 4.6 yr, 157 hip fractures), FT4 was not associated with fracture risk, whereas there was a borderline significant association between TSH (per SD decrease) and increased risk of hip fractures in men with normal TSH (0.55–4.78 mIU/L).^21^ We did not detect any association between thyroid hormone levels and hip fracture risk in a previous analysis of MrOS-Sweden with shorter follow-up time (median follow-up 9.8 yr, 127 hip fractures).^22^ In contrast, in the present study with longer follow-up and more incident hip fractures, we found an association between higher FT4 and increased hip fracture risk.

In the Cox proportional hazards regression analyses, among men with normal TSH, a borderline significant association was observed between serum TSH (per SD decrease) and an increased risk of hip fractures in model A, which lost significance after additional adjustment for total hip sBMD in the fully adjusted model (model B). However, as low normal TSH has been related to reduced BMD,^1^^,^^29^ it may be argued that BMD is a mediator and not a confounder in terms of the association between TSH and hip fracture risk. Furthermore, these findings are in some accordance with the results of the MrOS-US study, in which there was a borderline significant association between TSH (per SD decrease) and increased risk of hip fractures in men with normal TSH.^21^ In the Hunt cohort (16 610 women and 8595 men), TSH was not associated with hip fracture risk,^16^ whereas in a Danish register-based study, the risk of hip fractures was increased with each SD unit of TSH decrease in subjects with normal TSH at baseline.^18^ Additionally, lower TSH as well as higher FT4 was associated with increased risk of hip fractures in a pooled analysis of 13 prospective cohort studies.^19^ Although there is a strong negative correlation between FT4 and TSH levels, the main function of TSH is to regulate T4 secretion, whereas T4 and T3 exert the main effects on the peripheral targets. Our results, in conjunction with this understanding, suggest that FT4 could be a more relevant and considerably stronger marker of the bone effects of thyroid hormones as compared with TSH. Still, it seems reasonable to assume that measurements of both TSH and FT4 are needed to have a complete assessment of the risk of hip fractures.

In addition to the association with an increased risk of hip fractures, serum FT4 (per SD increase) in men was also associated with an increased risk of MOF. This association remained statistically significant after full adjustment for covariates and even after excluding men with serum TSH outside the reference range. There was also a tendency toward an increased risk of MOF in men in the highest FT4 quartile compared with men in the three lower FT4 quartiles, although this association was not statistically significant in the fully adjusted model. Whether there is an independent association between TSH or FT4 and the risk of MOF has previously not been evaluated in a study population consisting only of men, but a few studies in postmenopausal women and some studies of both men and women have indicated such an association.^15^^,^^18^ Furthermore, we found a significant association between FT4 as a standardized continuous variable and the risk of vertebral fractures in our total study population in the fully adjusted model (model B). However, this association lost statistical significance in the subanalysis that included additional adjustment for the time of serum sampling. In men with normal TSH, we could not detect any significant association between FT4 as a standardized continuous variable and the risk of vertebral fractures after full adjustment for covariates.

The present study has several strengths and weaknesses. Strengths are the large cohort of well-controlled elderly men as well as the long follow-up and the relatively high number of incident hip fractures. We had reliable fracture data based on computerized X-ray archives, enabling us to include only X-ray-verified fractures with information about the time and site of each fracture. Furthermore, all blood samples were drawn at baseline of the study. Thus, none of the blood samples were obtained on a clinical indication. However, the procedures for blood sampling were partly different in the Malmö and Gothenburg subcohorts of MrOS-Sweden, but we used MrOS site as a covariate in all analyses and, in addition, we performed a subanalysis in which we included the time of serum sampling as a covariate. We excluded men with levothyroxine treatment at baseline or a previous history of thyroid cancer; however, it was not recorded whether the men had a previous thyroid disorder. Moreover, medications were only recorded at baseline and therefore, some of the included men could have received levothyroxine treatment or antithyroid drugs that were initiated after baseline. As expected, some of the men died during the follow-up period, and we cannot exclude the possibility that survival bias could have affected our results. Another limitation is that self-reported questionnaires were used, and we cannot exclude that this could have resulted in an underestimation of the prevalence of smoking. Additionally, most measurements were only performed at baseline (thyroid hormones, sBMD, and covariates/confounders). Therefore, we cannot evaluate if there were changes in thyroid hormone levels across the study period. Finally, all participants were elderly Caucasian men, which may reduce the generalizability of our findings to other populations.

In summary, this is the study with longest follow-up time and highest number of incident hip fractures that has determined the associations between thyroid hormones and the risk of incident fractures in a study population consisting only of older men. We extend the previous knowledge by showing that higher serum FT4 is markedly associated with the long-term risk of hip fractures in older Swedish men. We also found an association between higher FT4 (per SD increase) and the risk of MOF both in the total study population and in men with normal TSH. In contrast, we did not find any association between serum TSH and the risk of fractures after full adjustment for covariates. Overall, our findings suggest that if FT4 levels are measured early, there is a possibility that preventive actions can be initiated when the risk of hip fractures is still moderate.

## Data Availability

The raw data used in the current study are restricted in order to protect participant privacy, as required by data protection acts in Sweden. Data can be made accessible by request for researchers after permission from the Swedish Ethics Review Authority.
